# How effective are trained dogs at alerting their owners to changes in blood glycaemic levels?: Variations in performance of glycaemia alert dogs

**DOI:** 10.1371/journal.pone.0210092

**Published:** 2019-01-15

**Authors:** Nicola J. Rooney, Claire M. Guest, Lydia C. M. Swanson, Steve V. Morant

**Affiliations:** 1 Animal Welfare and Behaviour Group, Bristol Veterinary School, University of Bristol, Bristol, United Kingdom; 2 Medical Detection Dogs, Greenway Business Park, Milton Keynes, United Kingdom; 3 Medicines Monitoring Unit, University of Dundee, Dundee, United Kingdom; Memorial University of Newfoundland, CANADA

## Abstract

**Aims:**

Domestic dogs are trained to a wide variety of roles including an increasing number of medical assistance tasks. Glycaemia alert dogs are reported to greatly improve the quality of life of owners living with Type 1 diabetes. Research into their value is currently sparse, on small numbers of dogs and provides conflicting results. In this study we assess the reliability of a large number of trained glycaemic alert dogs at responding to hypo- and hyper-glycaemic (referred to as out-of-range, OOR) episodes, and explore factors associated with variations in their performance.

**Methods:**

Routine owner records were used to assess the sensitivity and specificity of each of 27 dogs, trained by a single UK charity during almost 4000 out-of-range episodes. Sensitivity and positive predictive values are compared to demographic factors and instructors’ ratings of the dog, owner and partnership.

**Results:**

Dogs varied in their performance, with median sensitivity to out-of-range episodes at 70% (25^th^ percentile = 50, 75^th^ percentile = 95). To hypoglycaemic episodes the median sensitivity was 83% (66–94%) while to hyperglyaemic episodes it was 67% (17–91%). The median positive predictive value (PPV) was 81% (68–94%), i.e. on average 81% of alerts occurred when glucose levels were out of target range. For four dogs, PPV was 100%. Individual characteristics of the dog, the partnership and the household were significantly associated with performance (e.g., whether the dog was previously a pet, when it was trained, whether its partner was an adult or child).

**Conclusions:**

The large sample shows that the individual performance of dogs is variable, but overall their sensitivity and specificity to OOR episodes are better than previous studies suggest. Results show that optimal performance of glycaemic alert dogs depends not only on good initial and ongoing training, but also careful selection of dogs for the conditions in which they will be working.

## 1. Introduction

Hypoglycaemia is a common side effect of intensive insulin management amongst patients with Type 1 diabetes. It can be very distressing and presents risk of serious neurological and cardiovascular consequences (e.g., [1]), especially for patients who have lost the early warning signs of impending blood glucose changes. Unawareness is reported in 25% of Type 1 diabetes patients, increasing their risk of severe hypoglycaemic (low blood glucose) episodes 6-7-fold [2]. Fear of hypoglycaemia is very common [3] particularly related to night time hypoglcaemic episodes which are a potential cause of death. This fear can result in patients manipulating insulin levels, or “running their blood sugars high”, which can increase the risk of long-term deleterious consequences of hyperglycaemia (high blood glucose; [2]).

There are a variety of technologies available to patients, but one potentially valuable and non-invasive intervention is the glycaemia alert dog. Case studies and surveys [4] suggest that some pet dogs naturally respond to their owners’ hypoglycaemic state, and based on this, charities have started to train dogs to live with people with Type 1 diabetes. Similar to dogs trained to detect contraband, these dogs are conditioned to respond with specific alerting behaviours when their owners’ blood sugars fall outside a target range, known as an out-of-range (OOR) episode. This prompts the patient to test their blood glucose level, and to take appropriate action (e.g., insulin administration or eating) to retain appropriate glucose levels. While some organisations train all dogs to a generic range (see [5]), others train individual dogs to best meet the specific needs of the individual client [6].

At its best, a trained alert dog has the potential to vastly improve the quality of life of people living with Type 1 diabetes, allowing them to more tightly regulate their blood sugars and avoid the risks of both hypoglycaemic episodes and long-term health consequences of hyperglycaemia. However, there remains the possibility that having a trained dog simply produces a placebo effect with benefits no greater than those known to be associated with general dog ownership (see [4]). Since the usage of such dogs is growing, and false confidence in the dogs’ abilities could have detrimental consequences, it is vital that their true efficacy be assessed, and factors affecting performance identified.

There have been a growing number of studies of “Diabetic Alert Dogs” (see [7]). One small study showed that three dogs trained to work with their owners showed relatively low sensitivity (true positive rate) and specificity (true negative rate) when tested on remote skin samples [8]. A further study demonstrated that dogs have the potential to distinguish between perspiration samples from hypo- and eu-glycaemic states; six dogs were tested remotely and demonstrated sensitivities ranging from 50 to 87.5% and specificities of 89.6 to 99.7% [9]. Several case examples [10] and exploratory studies in both the USA [11,12] and UK [6], have documented that people believe they have significantly fewer hypoglycaemic episodes and improved Quality of Life after acquiring an alert dog [6]. Owner-reported data, supplied by 17 dog users, also supported survey results with the majority of patients’ routine blood records showing tightened glycaemic control after dog acquisition as compared to before.

A single recent study, however, has utilised blinded glucose monitoring records and questioned the universal value of alert dogs [5]. Comparing eight dogs trained by a variety of agencies, Los et al. (2016; [5]) reported average sensitivities (proportion of hypoglycaemic episodes correctly identified by the dogs) of only 36% over 45 hypoglycaemic episodes. More concerning is that they reported high levels of false alerting, resulting in average positive predictive values (PPVs; proportion of alerts that are correct) of only 12%. Thus, the dogs were deemed less reliable than the other monitoring systems tested. However, one must also consider that the equipment used to measure blood glucose is not 100% accurate or reliable, with different technologies producing varying time lags (e.g.,[13]).

The Los et al. (2016) study [5] also raises several questions:

- Although the dogs were reported to respond to hyperglycaemia as well as hypoglycaemia, alerts during times of high blood sugar were classified as incorrect, which may account for a significant proportion of the “false alerts”. Although agencies generally do not train dogs specifically to respond to levels above their owner’s target range, they report that many dogs naturally start doing so (and are usually rewarded for such responses). The reliability of dogs at alerting at time of high, as well as low, blood sugars needs to be examined to fully assess diabetic alert dog efficiency.
- The study includes a small and diverse sample of dogs, yet reports only mean sensitivity and PPV levels, rather than individual dog’s proficiency. A further study reports large inter-dog variability [6], the extent and causation of which remains untested. Trainers and instructors of diabetes dogs describe traits in individual dogs which they believe are associated with better performance (pers comm). They also describe the nature of the person’s diabetes (e.g., the speed with which blood sugars change), their lifestyle (e.g. busyness of the household and the consistency of their behaviour towards their dog), and their attitude towards their dog, (e.g., confidence in its ability), to affect the dog’s success at alerting their owner to OOR episodes. It is important to understand the factors associated with optimal performance in order to select dogs and train them in a way that maximises their alerting performance.
- A previous report has mentioned dogs alerting at times of rapidly falling, yet still within target range, blood glucose levels [6]. The existent of pre-alerting behaviour remains to be tested and was not explored in the study by Los et al. (2016; [5]). By varying the time window over which an alert is deemed correct, we can determine whether dogs are able to reliably “pre-alert”. This phenomenon is important, as if some dogs pre-alert to impending low or high blood sugars, their measured sensitivities will appear artificially low. The mechanisms by which they achieve pre-alerting abilities are also of importance to elucidate as they potentially could inform improvement in future technology.

The current study explores the variability in glycaemia alert dog response rates further, using dogs trained by a single agency, Medical Detection Dogs, the only programme in the United Kingdom that is accredited by Assistance Dogs UK for training hypoglycaemia alert dogs. This charity receives no state funding and has strict standards for both general obedience in public places and task-related performance. Dogs are initially trained at the charity headquarters using *in vitro* samples obtained from the dog’s prospective owner. Samples of breath (post-2014) or sweat (prior to 2014), taken when the person is in a hypoglycaemic state are paired with a food reward. Dogs are trained to respond to the samples with specific behaviours, such as licking the owner or fetching the owner’s blood testing kit [14]. The charity has gradually evolved to optimise training methods based on cumulative experience and potentially important changes occurred in 2014, before which dogs were trained to show specific a-priori chosen alerts, and after which individual dogs were trained to show the alert behaviours which they naturally favoured. This study represents the first large-scale evaluation of the efficacy of dogs trained before and after these protocol changes.

Medical Detection Dogs protocol stipulates, that after initial centred-based training, when trainee dogs are performing reliably to the future partner’s odour (after approximately 7 weeks), dogs are paired with that client, and *in situ* training continues. Prior to accreditation clients are required to keep records of all routine blood glucose tests (taken a minimum of six times per day) and also to test their blood glucose levels every time their dog shows an alerting behaviour. Although *in vitro* training and accreditation involves only hypoglycaemia, dogs often spontaneously alert to hyperglycaemia when matched with their owner [6; 15]. The charity recommends rewarding these spontaneous alerts, but with a smaller reward. Dogs may sometimes show “response alerts” performing the required behaviour only after their owner has conducted a blood test. Clients are generally advised to reward these, but since they do not represent a spontaneous alert, they are not included in any calculation of dog sensitivity.

To be accredited, dogs much achieve a level of 75% sensitivity (to hypoglycaemic samples) with less than 15% (PPV>85%) false alerts over a consistent period of three months, as well as pass a standardised “public access” test as required by Assistance Dogs UK. Once the partnership is accredited, the client is primarily responsible for rewarding their dog and hence maintaining its performance, but regular instructor support and visits are designed to assist with this. Each client provides approximately six weeks of blood test and dog alerting data annually to allow performance to be monitored and problems identified. It is these data (combined with pre-certification data, in some cases) which we use in the current study.

Using a sample of 27 dogs, trained by this single agency, and records of 4197 hypo- and hyper-glycaemic episodes, we test several hypotheses:

1. Individual dogs vary in the sensitivity and specificity of their responses;
2. Dogs show differing sensitivities to high and to low blood sugar levels;
3. Individual differences in performance are associated with specific traits which differ between dog-owner partnerships and with the time for which the dog has been certified;
4. Some dogs respond when blood sugars are rapidly changing before they reach an out-of-target range.

## 2. Materials and methods

This research was approved by University of Bristol Faculty of Health Sciences Research Ethics Committee Reference: 09/10NR03

### 2.1 Subjects

This study used client-dog partnerships from the UK’s largest organisation training glycaemia alert dogs: Medical Detection Dogs. After dogs were trained individually and in combination with their human partners, each client provided three continual months of blood records detailing every time the dog alerted, and routine tests, prior to being fully accredited. They subsequently provided six weeks of data each year. All dog-client partnerships which had provided these data in the last three years and were currently accredited were approached and asked for consent to use their data anonymously for this project. Of the 34 partnerships approached, 32 agreed.

### 2.2 Data extraction

The most recent 6–12 weeks of data on record was extracted from electronic or paper records (subsequently entered into a database). These were either the pre-accreditation data, the most recent annual data, or a combination of both. Individual days with fewer than six glucose readings were deemed inadequate to determine OOR episodes and were excluded from subsequent analyses. Five partnerships (2, 4, 16, 29 and 31) never exceeded this threshold, so are not described any further. Data detailed the time of each finger prick glucose test or glucose pump reading, taken both routinely and whenever the dog showed a behavioural alert.

Basic demographic details of each dog-owner partnership were also extracted: age, sex, the date of accreditation and whether the dog was a former family pet trained *in situ* by the charity or a procured dog trained in the charity’s training headquarters and allocated to the client. From these data we derived three binary variables: whether the client was a child or adult; whether the dog was accredited at the time of data collection and whether the dog was formerly owned by the client or family-or centre-procured, and the continuous variable time since accreditation (in years). These variables were each used to test for associations with dog efficacy.

### 2.3 Instructor ratings

Medical Detection Dog client-dog instructors were asked which factors they believed most affected the performance of the dogs. Their responses described eleven apparently distinct traits, grouped into two categories: 1) **Client Specific:** *Busyness of the household*, *Severity of client’s diabetes*, *Speed of client’s drop in blood glucose level*, *Willingness to reward the alerts*, *Confidence in dog’s ability*, *Ability to recognise the dog’s alerts*, *Consistency of client’s behaviour towards their dog*, *Level of communication with the instructor (including with records)*; 2) **Dog Specific:** *Dog’s motivation and enjoyment of the task*, *Strength of dog’s alert*, *Dog’s willingness to try new behaviours and “get it wrong*”.

The instructors most familiar with each dog-client partnership were then asked to rate the partnership on a scale of 1–10 for each of the eleven traits. For partnerships rated by more than one instructor (the maximum was 3), an average was calculated. The instructors also reported the number of people most usually living in each dog’s household.

Among these 12 sets of scores there were nine highly correlated pairs (rank correlation > 0.7), each involving either *Busyness of household*, *Ability to recognise dog’s alerts* or *Consistency of client’s behaviour*. In subsequent analyses we did not include these three scores, which were highly confounded with measures we considered to be more objective.

### 2.4 Classification of owners’ records

We classified each glucose reading as hypoglycaemic, euglycaemic (within target range) or hyperglycaemic according to the glucose range each client’s dog was trained to alert (Table 1). Each 24-hour period (6AM to 6AM) was divided into contiguous episodes when clients were deemed to be within, below, or above their target range. A new episode started when a glucose concentration was in a different range from the previous one and continued while concentrations remained in the same range. The number of out of range episodes varied between clients from 3 to 411. Measures of dog alert efficacy included sensitivity, calculated as the proportion of OOR episodes (low and high separately) during which at least one alert was recorded, and positive predictive value (PPV), calculated as the proportion of recorded alerts at which the blood glucose was OOR (either low or high). Clients had recorded “response alerts” which occurred only after they had confirmed their blood glucose, and hence could potentially have been a response to the owner’s behaviour rather than odour were eliminated from analyses.

**Table 1:** Details of partnerships and blood glucose records from each of the 27 partnerships providing sufficient data for analysis (i.e., providing at least six glucose readings per day).

Partnership Number	Data collected after accreditation	Own dog	Child	Target Range (mmol/L)			Blood Glucose Records								
							All			High			Low		
					Tests per day	Routine tests per day	OOR episodes	Alerted (sensitivity)		OOR episodes	Alerted		OOR episodes	Alerted	
					Mean	Mean	N	N	%	N	N	%	N	N	%
1	Yes	No	No	4.5–15.0	23.1	17.6	411	242	58.9	267	156	58.4	144	86	59.7
3	No	No	Yes	4.6–11.0	15.4	5.7	251	219	87.3	158	137	86.7	93	82	88.2
5	Yes	No	No	5.0–10.0	9.7	4.0	102	84	82.4	72	59	81.9	30	25	83.3
6	Yes	No	Yes	5.5–12.7	8.3	4.8	46	37	80.4	11	8	72.7	35	29	82.9
7	No	Yes	No	5.0–11.9	9.0	3.6	290	274	94.5	199	191	96.0	91	83	91.2
8	No	No	No	4.6–11.9	10.1	6.4	361	252	69.8	64	50	78.1	297	202	68.0
9	Yes	No	No	4.5–8.5*	8.3	3.8	285	211	74.0	129	56	43.4	156	155	99.4
10	No	No	Yes	5.0–12.0	10.8	8.4	192	86	44.8	68	9	13.2	124	77	62.1
11	No	No	Yes	5.5–10.0	14.1	12.0	82	25	30.5	53	0	0.0	29	25	86.2
12	No	No	No	5.0–12.0	16.3	12.5	213	196	92.0	81	74	91.4	132	122	92.4
13	Yes	No	Yes	5.0–13.5	9.4	8.1	17	4	23.5	10	0	0.0	7	4	57.1
14	No	No	No	4.7–8.5*	7.3	5.1	283	183	64.7	164	103	62.8	119	80	67.2
15	Yes	No	No	4.5–9.7	13.3	4.8	151	105	69.5	106	71	67.0	45	34	75.6
17	No	No	No	5.0–15.0	9.7	6.4	158	124	78.5	20	10	50.0	138	114	82.6
18	Yes	No	Yes	5.5–14.9	7.1	6.1	22	2	9.1	11	0	0.0	11	2	18.2
19	No	No	No	4.5–14.0	19.6	13.6	96	93	96.9	4	3	75.0	92	90	97.8
20	No	Yes	Yes	4.5–12.0	9.6	2.2	28	28	100.0	15	15	100.0	13	13	100.0
21	No	Yes	No	4.5–12.0	8.8	6.1	192	106	55.2	96	20	20.8	96	86	89.6
22	No	Yes	No	4.5–12.0	7.6	6.8	13	2	15.4	8	0	0.0	5	2	40.0
23	Yes	No	No	5.5–14.9	6.6	0.3	7	7	100.0	2	2	100.0	5	5	100.0
24	Yes	No	No	5.0–12.0	6.4	4.8	48	24	50.0	18	3	16.7	30	21	70.0
25	Yes	No	Yes	4.5–12.0	6.0	1.0	3	3	100.0	2	2	100.0	1	1	100.0
26	Yes	No	No	4.7–8.0*	6.7	5.3	120	29	24.2	76	0	0.0	44	29	65.9
27	Yes	No	No	4.5–11.0	7.1	4.1	78	76	97.4	50	48	96.0	28	28	100.0
28	No	No	No	4.5–12.0	7.8	4.7	138	90	65.2	99	55	55.6	39	35	89.7
30	Yes	Yes	No	6.0–16.0	8.4	6.4	73	41	56.2	33	23	69.7	40	18	45.0
32	Yes	No	No	4.5–12.9	12.7	7.5	112	107	95.5	76	73	96.1	36	34	94.4

*these very narrow target ranges with hyperglycaemia classified above 8 are unusual and not recommended by the charity

### 2.5 Statistical analysis

Sensitivity and positive predictive values are binomial proportions and were modelled using logistic regression. The demographic variables of whether the dog was accredited at the time of data collection, whether the client was adult or a child, and whether or not the dog was initially family-owned were categorical. Instructor ratings for each of the eight traits (on a scale from 0 to 10), size of household (in numbers), and length of time since accreditation (designated as 0 for pre-accreditation partnerships) were treated as continuous variables. These were assessed individually as potential predictors of sensitivity and PPV, except that effects associated with accreditation and time since accreditation were estimated jointly. Results are presented as odds ratios (per unit change for continuous predictors). We regard these analyses as exploratory, rather than hypothesis testing, and therefore no adjustments have been made to preserve Type 1 error rates.

In order to explore evidence of pre-alerting behaviours, we explored how many of the apparently incorrect alerts were followed by an OOR episode either within 20, 30, 40, 50 or 60 minutes.

## 3. Results

The dogs were predominantly Retriever first generation crosses (e.g. Golden x Labrador Retriever or Curly Coat x Labrador Retriever; n = 12) and Labrador Retrievers (n = 6), but also included a Bichon Frise (1), Cocker Spaniels (2), a Poodle (1), a Poodle cross (1), a Staffordshire Bull Terrier (1), an English Springer Spaniel (1) and a Yorkshire Terrier (1). Dog and owner had been accredited for between 0.2 and 3.2 years (median 1.5 years, interquartile range 0.7 to 2.0) at the time of data collection. Twelve of the clients had provided data prior to being certified (and had subsequently passed certification requirements), 13 provided annual post-accreditation data (for one client this spanned several years), and for one client a combination of both data types (client 19) was used to provide sufficient data to analyse (Table 1). Eight dogs were partnered with a child (under 18 years old) and five were the client’s own dog and had lived with them prior to being trained by Medical Detection Dogs charity. The clients were 11 men/boys and 16 women/girls.

**Fig 1:**
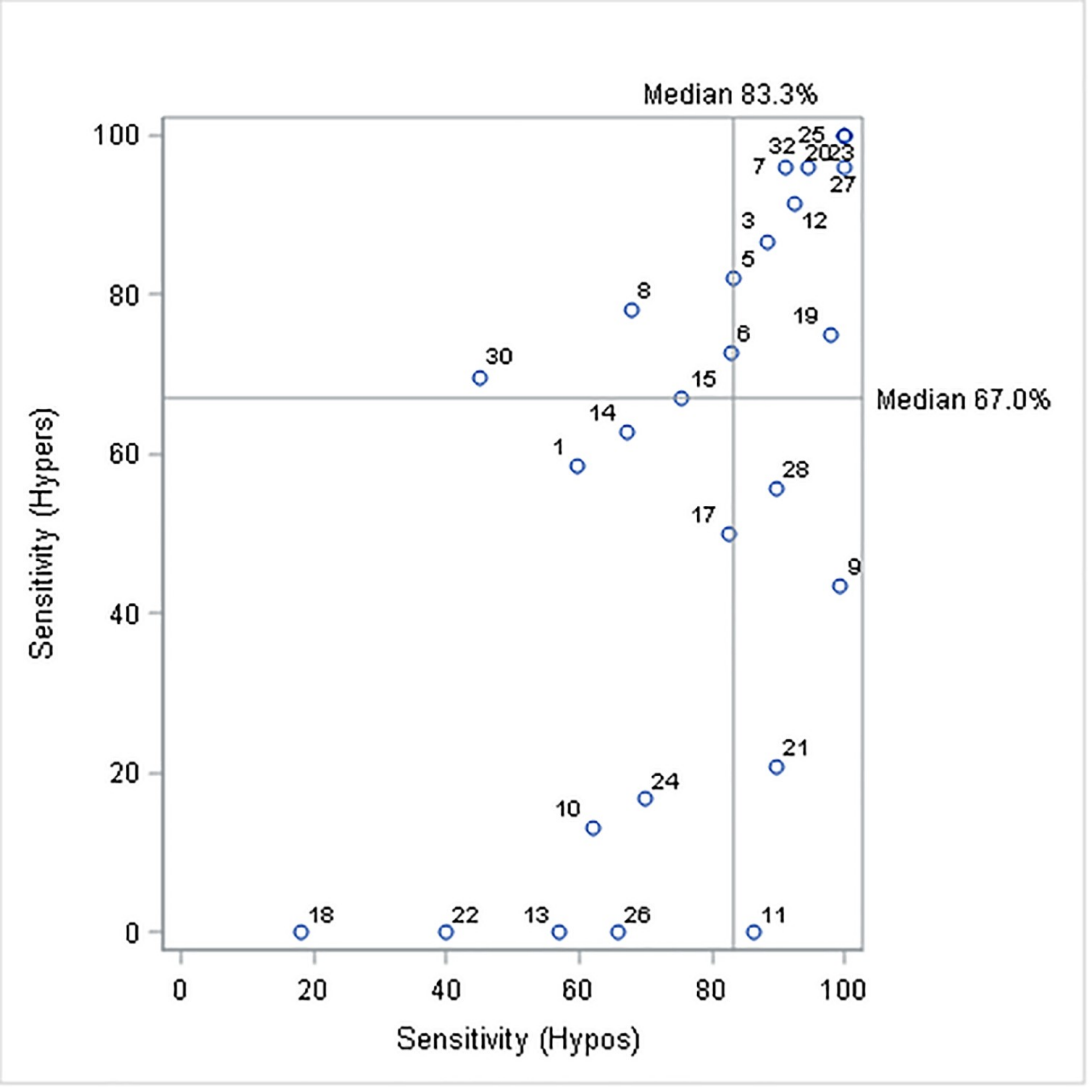
Mean sensitivity to low (hypoglycaemic, Hypos) and high (hyperglycaemic, Hypers) out-of-range episodes, for all 27 partnerships who provided consent and more than six reading on at least one day. Each circle represents a partnership and the horizontal and vertical lines show the population medians for sensitivity to hypo- and hyper-glycaemic episodes respectively.

Dogs varied in their performance, with median sensitivity to OOR episodes at 70% (median, 25^th^ percentile = 50%, 75^th^ percentile = 95%), and with all but six dogs having values of at least 50%. Median sensitivity to hypoglycaemic episodes was 83% (66%-94%), while to hyperglyaemic episodes it was 67% (17%-91%) (Fig 1). Considering individual dogs, sensitivity to low OOR levels (hypoglycaemia) was often very different to that for high OOR levels (hyperglycemia), although there was a moderate rank correlation between the two (Fig 1; Spearman’s rho = 0.72, p<0.001).

**Fig 2:**
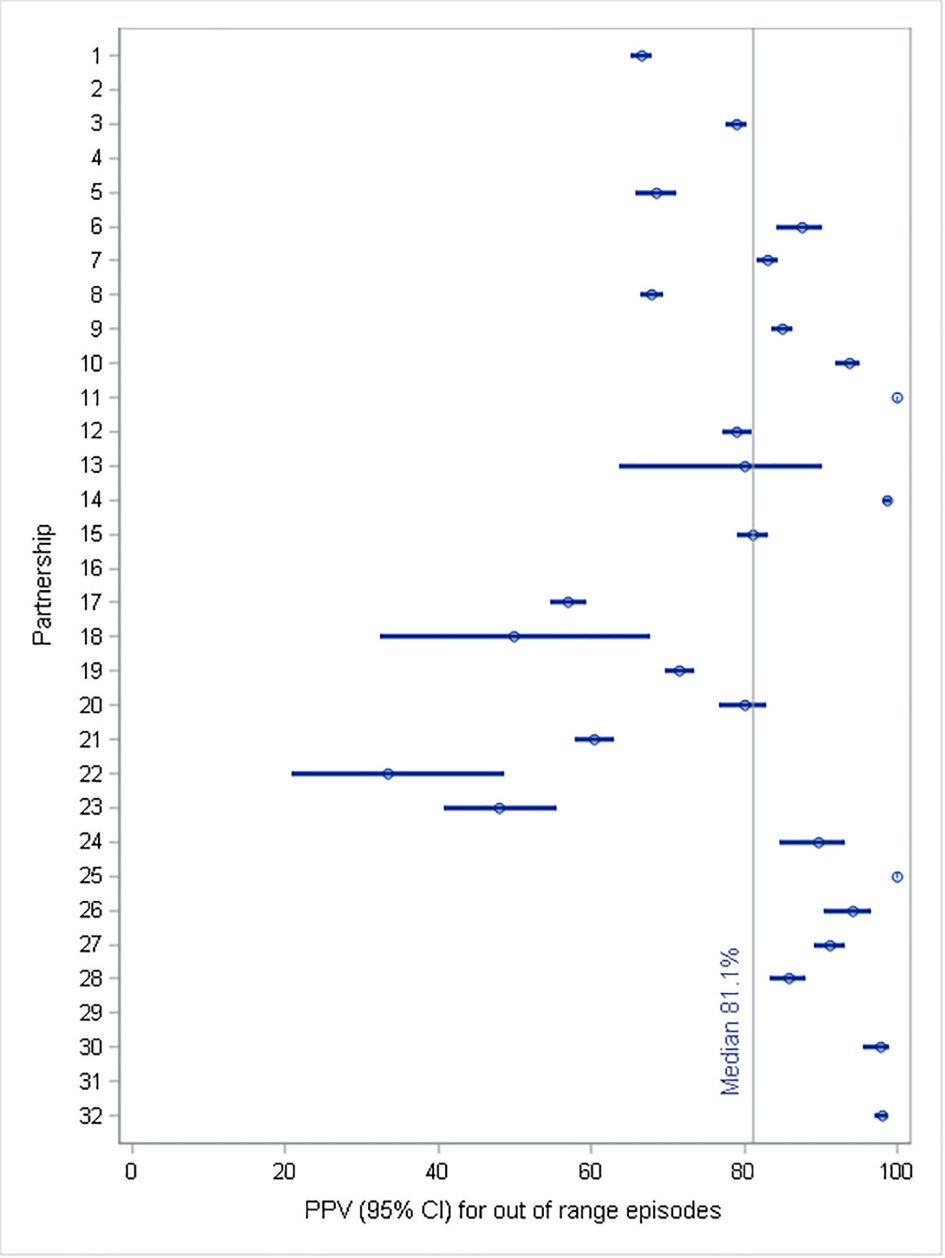
Mean Positive Predictive Values (PPV), with confidence intervals for all alerts for 27 partnerships who provided consent and more than six readings on at least one day. The vertical line represents the population median for PPV ie proportion of alerts were classified as correctly indicating an OOR episode.

The median positive predictive value was 81% (68%-94%), so for half of the partnerships 81% of more of alerts occurred when glucose levels were out of target range. Only two dogs were apparently incorrect in over half of their alerts. Four dogs had a PPV of 100% (Fig 2). There was no significant correlation between the PPVs and sensitivity to either low or high OOR episodes (p = 0.50 and p = 0.78, respectively).

**Fig 3:**
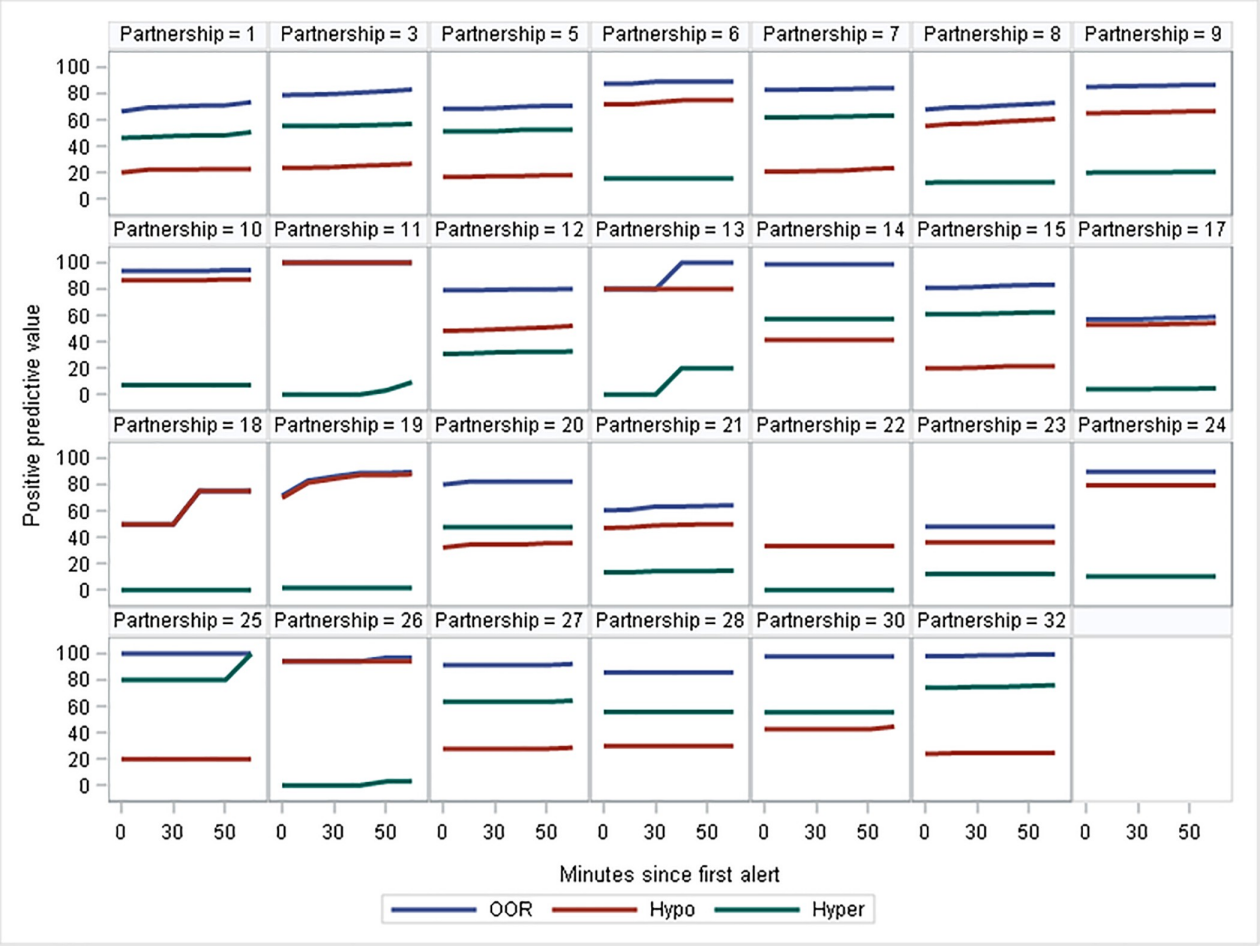
Individual plots to show each partnership’s proportion of alerts which were classified as correctly indicating an OOR episode (Postive Predictive Value, PPV) when glucose levels up to 50 minutes following the alert were taken into account.

**Fig 4:**
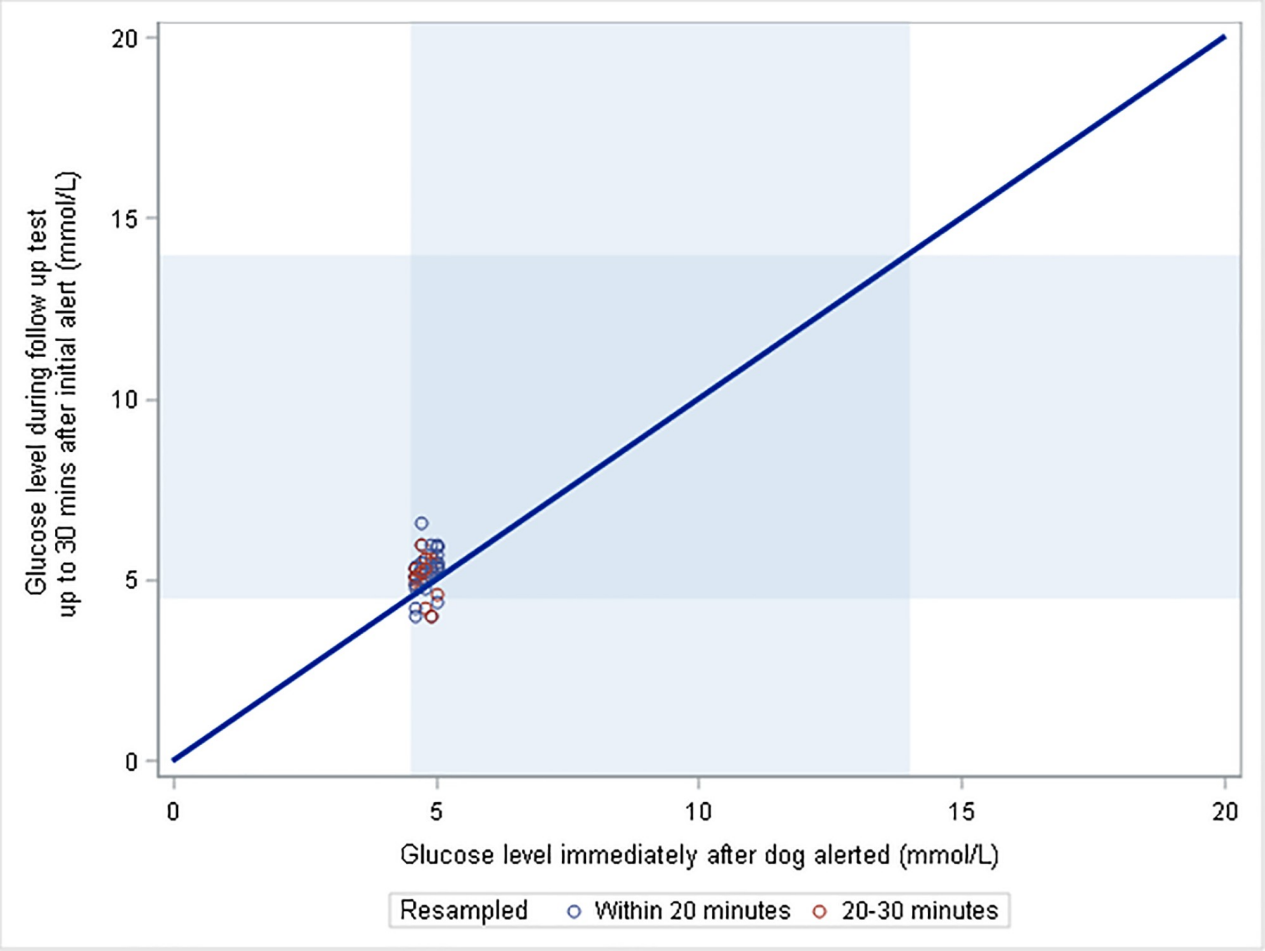
Glucose values for partnership 19, whose dog showed an indication of pre-alerting behaviour, for all apparent incorrect alerts (i.e. when the glucose value at an alert fell within the shaded target range on the horizontal axis) and for re-tests carried out within 30 minutes (the vertical axis). The diagonal line represents no change in glucose between the alert and re-test.

Newly accredited dogs had better sensitivity to hypoglycaemic episodes than unaccredited dogs (OR 3.14 (1.88, 5.23)), but sensitivity was generally lower in dogs that had been accredited longer (OR 0.4 (0.30,0.54) per year; Table 2). Dogs generally had poorer sensitivity to hyperglycaemic episodes (OR 0.37 (0.27,0.52)), but sensitivity was higher in those that had been accredited for longer (OR 1.63 (1.34,1.98 per year; Figs 3 and 4). Newly accredited dogs had better PPV than unaccredited dogs (OR 2.21 (1.63,2.98)), and this too was lower in those that had been accredited for longer (OR 0.64 (0.55,0.76) per year). Dogs which were originally household pets (i.e., client’s own dogs, of which there were five) were more sensitive to high, but not low, blood sugars than were dogs procured by the charity and then placed with the client (Table 2). Those partnered with children (as compared to adults) were significantly less sensitive to both high and low blood sugar levels but showed significantly higher PPVs; however,there were only eight such partnerships, making this finding tentative (Table 2).

**Table 2:** Odds ratios for sensitivity and positive predictive value (PPV) by potential predictors.

	Sensitivity				Positive predictive value (PPV)	
	Hypoglycaemic episodes		Hyperglycemic episodes			
	OR (95% CI)	p value	OR (95% CI)	p value	OR (95% CI)	p value
Categorical						
Accredited at time of data collection^1^	3.14 (1.88, 2.23)	<0.001	0.37(0.27, 0.52)	0.001	2.21 (1.63, 2.98)	<0.001
Client's own dog	1.30 (0.92, 1.85)	0.138	1.65 (1.28, 2.13)	< .001	1.01 (0.84,1.22)	0.908
Partnered with a child	0.74 (0.56, 0.98)	0.038	0.62 (0.49, 0.79)	< .001	1.55 (1.27, 1.88)	< .001
Continuous^2^						
Time since accreditation (per year) ^1^	0.40 (0.30, 0.54)	<0.001	1.63 (1.34, 1.98)	<0.001	0.64 (0.55, 0.76)	<0.001
Severity of client’s diabetes	1.15 (1.06, 1.24)	< .001	1.13 (1.07, 1.20)	< .001	0.98 (0.94, 1.02)	0.311
Speed of drop in client’s glucose	1.43 (1.30, 1.57)	< .001	1.05 (0.98, 1.12)	0.171	1.03 (0.98, 1.09)	0.238
Client’s level of communication with the instructor (including with records)	1.54 (1.36, 1.74)	< .001	1.14 (1.04, 1.24)	0.005	1.05 (0.96, 1.15)	0.268
Size of household	0.89 (0.83, 0.96)	0.002	0.87 (0.81, 0.94)	< .001	1.08 (1.02, 1.14)	0.008
Client’s willingness to reward dog’s alerts	1.49 (1.34, 1.66)	< .001	0.85 (0.76, 0.95)	0.004	1.22 (1.14, 1.31)	< .001
Confidence in dog’s ability	1.42 (1.31, 1.55)	< .001	1.14 (1.04, 1.24)	0.003	1.11 (1.05, 1.18)	< .001
Dog’s motivation and enjoyment of the task	1.42 (1.32, 1.54)	< .001	0.94 (0.87, 1.02)	0.132	1.13 (1.07, 1.19)	< .001
Strength of dog’s alert	1.25 (1.19, 1.31)	< .001	1.11 (1.05, 1.18)	< .001	1.08 (1.04, 1.12)	< .001
Dog’s willingness to try new behaviours and “get it wrong”	1.28 (1.21, 1.36)	< .001	1.18 (1.13, 1.24)	< .001	0.92 (0.89, 0.96)	< .001

^1^ Estimates for Accredited and Time since accreditation from the same multivariate model. All other analyses were univariate.

^2^ Odds ratios per year or unit change in score

There were a number of the partnership traits which were associated with better alert performance (see odds ratios in Table 2). Specifically, increased sensitivity was associated with dogs scoring higher for *Strength of dog’s alert*, *Willingness to try new behaviours and “get it wrong” and Dog’s Motivation and enjoyment of the task* (only sensitivity to hypoglycaemic episodes was significantly affected). Increased sensitivity was also associated with the client-based factors of higher *Level of communication with the instructor*, higher *Severity of client’s diabetes*, greater *Clients’ willingness to reward dog’s alerts*, smaller *Size of household*, *and* higher *Speed of drop in client’s glucose* (only hypoglycaemia sensitivity was significantly affected),

Higher PPV (fewer false responses) was positively associated with *Client’s willingness to reward dog’s alerts*, *Confidence in their dog’s ability*, *Size of household*, and with dogs that were rated higher for *Motivation and enjoyment of the task*, *Strength of dog’s alert*, and *Willingness to try new behaviours and get it wrong*” (Table 2).

The graphs comparing the PPV when the timeframe over which a response was deemed correct was varied from 0 to 60 minutes prior to an OOR episode, show little variation in slopes, which were generally shallow (i.e., PPV at 50 mins are generally very similar to at 0 mins), and, hence, there is little evidence of dogs responding ahead of OOR tests (Fig 3). One dog appeared to show a pronounced increase in PPV when previously incorrect responses were tested 30 minutes later (Fig 3: Partnership 19). However, Fig 4 illustrates that most of those previously in range values were extremely close to the hypoglycaemic range. For two other partnerships (13 and 18), there was an increase in PPV when samples taken 40 minutes after the alert were included, but there was no trend prior to 40 minutes.

## 4. Discussion

Analysis of owner-recorded data shows that most glycaemic alert dogs trained by the Medical Detection Dogs charity operate with considerable sensitivity, alerting their owners to a large proportion of out-of-range (OOR) episodes. However, even within a single agency with strict performance criteria, dogs vary greatly in measures of their performance. Median sensitivity to all OOR episodes (both hypo- and hyper-glycaemic episodes) was 70%. However, if only sensitivity to low glucose levels (to which the dogs are formally trained to respond) is considered, the median was 83% (i.e. for half the partnerships, dogs alerted owners to a minimum of 83% of hypoglycaemic episodes). These rates are considerably higher than those reported by Los et al. (2016: 36%; [5]), and above the 75% required for initial accreditation, suggesting that sensitivity of most dogs is maintained post-accreditation, and that dogs are generally very reliable in alerting their owners to OOR glucose levels. However, there were ten dogs whose sensitivity at the time of data collection was below the 75% criteria required for accreditation, and would hence according to charity policy have received extra training and support from their instructors. While there were many dogs which showed very high sensitivities at both ends of the OOR glucose spectrum, others performed better at one end compared to the other, resulting in only a moderate rank correlation between a dog’s sensitivity to hypo- and to hyperglycaemia (Spearman’s rho = 0.72). In particular, lower sensitivity to hyperglycaemia was common (nine dogs detected less than 50% of high glucose level episodes). This is unsurprising given that dogs are not trained to respond to hyperglycaemia although many appear to do so naturally and are generally rewarded for such responses, as long as the glucose level is outside the owner’s target glucose range.

There were, however, a small number of dogs whose performance at the time of data collection was relatively poor; three dogs showed sensitivities to hypoglycaemia below 50% and two showed PPVs below 50%. This may be a result of the client currently experiencing additional health problems or issues with their diabetes management, or may be a sign of potential dog training issues, which require remedial attention. The variation in performance also shows that, although all dogs are trained to an equivalent high standard initially (75% sensitivity), once working in the home, environmental factors may lead to differences in performance for a substantial number of them.

What may seem surprising is that those dogs which were accredited for longer and hence had been operational for more time were significantly more sensitive to high blood glucose levels, but significantly less sensitive at low glucose levels, and showed lower PPV than those trained more recently. This, combined with the finding that the five dogs which were originally the clients’ family pets showed higher sensitivity to hyperglycaemia episodes, suggests that hyperglycaemic detection ability may be something which is facilitated by a more established dog-owner relationship. It is possible that when responding to high blood glucose levels, dogs rely more on behavioural cues, detection of which is facilitated by greater familiarity with the human, while low blood sugars are detected predominantly using olfactory cues. However, since the scent of hyperglycaemia is detectable to humans (e.g. [16]), this in unlikely the sole explanation. Another explanation may be that although dogs exit training responding reliably to hypoglycaemic episodes, reinforcement to alerts in the home environment may be inconsistent, especially by owners suffering rapid drops and experiencing a reduced ability to respond, and/or cognitive impairment during episodes. The lack of consistent and appropriate rewarding may, with time, reduce the dogs’ sensitivity and specificity to hypoglycaemic episodes. While initial training (using remote samples prior to pairing with a client) does not include hyperglycaemic OOR samples, once operational, and working with the client, dogs receive rewards for responding at times of high glucose levels. Hyperglycaemic episodes have a potentially larger glycaemic range, the upper limit being infinite. Therefore, in contrast to hypoglycaemia, dog alerts may be more often and more reliably rewarded; hence, longer established partnerships perform better. This may explain why sensitivity to low levels is greater in dogs more recently trained, while for high levels the reverse is true. Additional efforts to retain sensitivity to hypolycaemia are required, and the results also highlight the importance of longer-term monitoring of performance and provision of supplementary training to operational dogs as required.

Dogs partnered with children were recorded to generally show lower sensitivity, although only eight out of the 27 partnerships were children. This difference may be a result of dogs generally not attending school with their child partners, and hence being unable to respond to all OOR episodes. Schools represent a logical challenge for alert dogs, with many children present, some of whom may have fears or allergies to dogs, so most dogs stay at home during school hours. Despite instructions to clients, often the records did not reliably show when the dog was absent from the child, and hence some of the child vs. adult differences in OOR sensitivity may be explained by dogs being absent during some of the OOR episodes. However, this lower sensitivity to children clients warrants further research.

Analysis of PPV shows that of the recorded alerts a median of 81% occurred during OOR episodes. This value is considerably higher than the 12% reported by Los et al. in 2016 [5]. In the current study, however, values ranged from 33 to 100%, with only two dogs, one of which was still in training, showing values less than 50%. PPV values tended to be higher in those dogs which were partnered with a child. For those dogs, the trained response is more complex, as it generally involves leaving the child and approaching a parent to show a trained behaviour. It would appear that dogs need to be more certain in order to produce this response and, hence, false alerts are less common.

Even when taking into account the time since accreditation, whether the dog was originally a family pet and whether the client is a child, significant variation in the efficacy of the trained dogs remained. The instructors reported a range of factors in the client, the dog, and the household which they believed contributed to this variation. Although several of these factors were inter-correlated, there were eight which were correlated to another at less than 0.7, hence one variable accounted for less than 50% of the variation in the other. When instructors rated these factors, they were each found to be significantly associated with either sensitivity or PPV, or both, as had been measured independently from client records. Hence, there are characteristics in the both dog and the human partner which are conducive to a more effective partnership, as has been shown for other working dog disciplines (e.g., [17,18]), and selection based upon these factors may facilitate better performing dogs in the future.

Interestingly, clients with more severe diabetes and quicker drops in glucose levels (as rated by their instructors in *Severity of client’s diabetes and Speed of drop in client’s glucose)* tended to have dogs performing with greater sensitivity (especially to low glucose levels). It is possible that the charity either deliberately selected dogs with better potential to work with these more vulnerable patients, or that dogs living in such situations learn to function better. Larger households were associated with lower sensitivity (more missed OOR episodes), yet higher PPV. This may be similarly explained to the differences between children and adults, as large families are more likely to contain children and may equally result in more time when the dog is separated from the human partner due to more potential distractions. Clients who had high *Levels of communication with the instructor* (including keeping thorough records) tended to have more sensitive dogs, likely as they took advice from the instructors and clear communication facilitated owners working to overcome problems before dog performance was affected. The dogs of owners who instructors scored higher for *Confidence in their dog’s ability* and those with higher *Willingness to reward dog’s alerts* appeared to maintain both a high level of correct responding and a low level of incorrect alerts. This highlights the importance of ongoing training in maintaining a well-functioning working animal (e.g.[19, 20]) and suggests the importance of appropriate selection and training of clients, as well as dogs.

The dogs which were recorded to perform the best, in terms of both sensitivity and PPV, tended to be those rated highest for *Motivation and enjoyment of the task* and *Strength of dog’s alert*. These traits have been shown to be important to search dog success (e.g., [17]), and developing methods to accurately predict and fine-tune these traits will prove integral to further improving diabetes alert dogs’ performance. An interesting trait described by instructors, and found to be significantly linked to both increased sensitivity and decreased PPV, was a dog’s W*illingness to try new behaviours and “get it wrong”*. During training, both initial and ongoing, dogs are often required to learn new behaviours and responses, and instructors describe some dogs as being conservative and reluctant to attempt new tasks, while others are more likely to offer new behaviours and to respond positively to cues which are unfamiliar. The latter type of dog would appear to produce a more sensitive trained dog, but one which also may potentially show more false alerts. We hypothesise that this trait may correlate with positive cognitive bias which has been described in dogs and other species (e.g., [21]), while a more pessimistic or risk-averse animal may score lower on the *Willingness to try new behaviours and “get it wrong”* trait. Cognitive biases have been shown both to be affected by temperamental characteristics [22] and modified by the living environment [23]. By further understanding the importance of, and ways to measure, this trait, it may be possible to select better dogs, and to modify training to facilitate greatest sensitivity and specificity of response. Further research into this area has great potential for understanding and improving alert dog efficacy.

It is often believed by clients and some trainers that some dogs show “pre-alerting behaviour” in which they alert their owners, yet when blood glucose levels are tested, they are still inside the target range; however, at subsequent testing (20–60 minutes later), the blood glucose levels have rapidly changed. Our data show little, if any, evidence of this phenomenon. On occasions when dogs are deemed incorrect in their alerting behaviours, glucose levels recorded in the next 20 or even 60 minutes are seldom OOR; hence, PPV is not greatly increased if these retests are taken into account. Our data showed one exception, partnership 19, for whom a large proportion of incorrect alerts were seen to be OOR 30 minutes following the alert. However, further examination of these data (as shown in Fig 4) show that such occasions generally occurred when the initial glucose test was very close to an OOR value, and, hence, the apparent pre-alerting behaviour may simply be explained by the dog generalising its behaviour to marginal values. While this study gives no evidence to support the phenomenon of pre-alerting, testing relied on owners having re-tested their blood glucose levels within a 20–60 minute window after an apparent false alert, which occurred relatively infrequently. To fully rule out the existence of pre-alerting behaviours, a targeted experimental design in which ideally glucose levels are monitored continually (e.g. using Continuous Glucose Monitoring) would be required. Since pre-alerting behaviour, if it exists, would imply that (some) dogs could respond more quickly than current machines, the phenomenon could be very valuable to understand, in order to inform technological advances.

Overall, the sensitivity and specificity of the dogs in responding to OOR episodes in this study were considerably higher than those reported in the study by Los et al. (2016; [5]), which likely reflects the rigorous training and accreditation procedures of this well-established charity. In comparison to Los et al. [5], we studied 27 as compared to eight dogs, and over 4000 (vs. 45) OOR episodes, and, hence, provide a more powerful study of the potential efficacy of diabetes alert dogs. It is important to acknowledge that all these data were provided by clients, and there remains the possibility that they excluded some less favourable records. However, since the original purpose of providing data was to improve their individual dogs’ training and the consent for research was only given post-hoc, it is reasonable to assume that the majority of these data were authentic. There is also the possibility that some OOR episodes were missed since glucose monitoring was not continuous. Dogs form just one part of a patient’s diabetes management plan, and clients still monitor blood glucose levels throughout the day, at meal times, and when glucose level changes are suspected. It is therefore unlikely that any severe episodes were missed. However, the possibility of some false negatives remains and further study with professional large-scale training establishments such as Medical Detection Dogs is required, using owner-independent measures to record both blood sugar and dog behaviour.
